# Peri-domestic entomological surveillance using private traps allows detection of dengue virus in *Aedes albopictus* during an autochthonous transmission event in mainland France, late summer 2023

**DOI:** 10.2807/1560-7917.ES.2024.29.36.2400195

**Published:** 2024-09-05

**Authors:** Barbara Viginier, Raphaëlle Klitting, Clémence Galon, Violaine Bonnefoux, Christophe Bellet, Albin Fontaine, Élise Brottet, Marie-Claire Paty, Armelle Mercurol, Nathalie Ragozin, Sara Moutailler, Gilda Grard, Xavier de Lamballerie, Frédérick Arnaud, Maxime Ratinier, Vincent Raquin

**Affiliations:** 1 EPHE, Université PSL, INRAE, Universite Claude Bernard Lyon1, IVPC UMR754, F-69007, Lyon, France; 2 National Reference Center for Arboviruses, Inserm-IRBA, Marseille, France; 3 Unité des Virus Émergents (UVE: Aix-Marseille Univ, Università di Corsica, IRD 190, Inserm 1207, IRBA), Marseille, France; 4 Anses, INRAE, Ecole Nationale Vétérinaire d’Alfort, UMR BIPAR, Laboratoire de Santé Animale, Maisons-Alfort, France; 5 Entente Interdépartementale Rhône-Alpes pour la démoustication (French public mosquito control organisation), Chindrieux, France; 6 Institut de Recherches Biomédicales des Armées (IRBA), Unité de virologie, Marseille, France; 7 Santé publique France (French National Public Health Agency), Lyon, France; 8 Santé publique France (French National Public Health Agency), Saint-Maurice, France; 9 Agence Régionale de Santé Auvergne-Rhône-Alpes (French Regional Health Agency), Lyon France

**Keywords:** Arbovirus, Vector, Mosquito, *Ae. albopictus*, dengue, Surveillance, Autochthonous, Epidemiology

## Abstract

While locally-acquired dengue virus (DENV) human infections occur in mainland France since 2010, data to identify the mosquito species involved and to trace the virus are frequently lacking. Supported by a local network gathering public health agencies and research laboratories, we analysed, in late summer 2023, mosquitoes from privately-owned traps within a French urban neighbourhood affected by a dengue cluster. The cluster, in Auvergne-Rhône-Alpes, comprised three cases, including two autochthonous ones. Upon return from a recent visit to the French Caribbean Islands, the third case had consulted healthcare because of dengue-compatible symptoms, but dengue had not been recognised. For the two autochthonous cases, DENV-specific antibodies in serum or a positive quantitative PCR for DENV confirmed DENV infection. The third case had anti-flavivirus IgMs. No DENV genetic sequences were obtained from affected individuals but *Aedes albopictus* mosquitoes trapped less than 200 m from the autochthonous cases’ residence contained DENV. Genetic data from the mosquito-derived DENV linked the cluster to the 2023–2024 dengue outbreak in the French Caribbean Islands. This study highlights the importance of raising mosquito-borne disease awareness among healthcare professionals. It demonstrates *Ae. albopictus* as a DENV vector in mainland France and the value of private mosquito traps for entomo−virological surveillance.

**Key public health message**

**What did you want to address in this study and why?**

Dengue virus (DENV) is transmitted to humans by mosquitoes of the *Aedes* genus, which act as vectors. When dengue affects an area, identifying the mosquito species transmitting the virus can inform mosquito control and prevent virus spread. Upon detecting a localised cluster of dengue cases in mainland France, a country which is not endemic for dengue, we collected mosquitoes from nearby privately-owned traps, to identify their species and analyse DENV.

**What have we learnt from this study?**

One trap yielded infected mosquito specimens belonging to the Asian tiger mosquito (*Aedes albopictus*) species. Results of the genetic analysis of the DENV strain in the mosquitoes linked the cluster to a dengue outbreak in the French Caribbean Islands. One case who had recently visited this area, had experienced symptoms compatible with dengue upon return to mainland France and had consulted healthcare; despite this, dengue had not been recognised.

**What are the implications of your findings for public health?**

This study shows that mosquitoes from private traps can be an effective tool for dengue surveillance, further fostering community engagement to address this disease. The findings also provide more evidence for *Aedes albopictus* being a DENV vector in Europe. Entomo−virological surveillance as well as raising awareness about mosquito-borne diseases among health professionals are key to improve the understanding of dengue outbreaks and their control.

## Background

Dengue is the most important mosquito-borne viral disease worldwide. A study published in 2013 estimated that 390 million dengue virus (DENV) infections (including asymptomatic ones) had occurred globally in 2010 [1]. Between 2000 and 2019, the amounts of dengue cases recorded by the World Health Organization (WHO) increased ca 10-fold [2]. Then, following a slight decrease in 2020–2022, a new upsurge of cases was noted in 2023 [2].

Although dengue is not endemic in continental Europe, DENV-infected travellers coming from dengue affected areas regularly arrive there. In the European Union/European Economic Area and United Kingdom (EU/EEA and UK), annual numbers of travel-associated dengue cases fluctuate with time. Except for 2021, the reported numbers of such cases per year since 2012 generally surpass 1,000 [3], reaching a maximum of 4,900 for the year 2023 [4]. Nevertheless, because infections can frequently be asymptomatic − ca 50% according to a meta-analysis [5] −, reports likely underestimate the true number of imported dengue cases to the EU/EEA and UK each year.

When environmental conditions are favourable, and DENV vectors are present in the European location of their arrival, viraemic travel-associated cases can occasionally pass on DENV to people living at this location, leading to limited clusters. Since 2010, when autochthonous cases were first reported from France and Croatia [6,7], 297 autochthonous cases have been identified in Europe, mainly in Italy, Spain and mainland France up to October 2023 [8].

**Figure 1:**
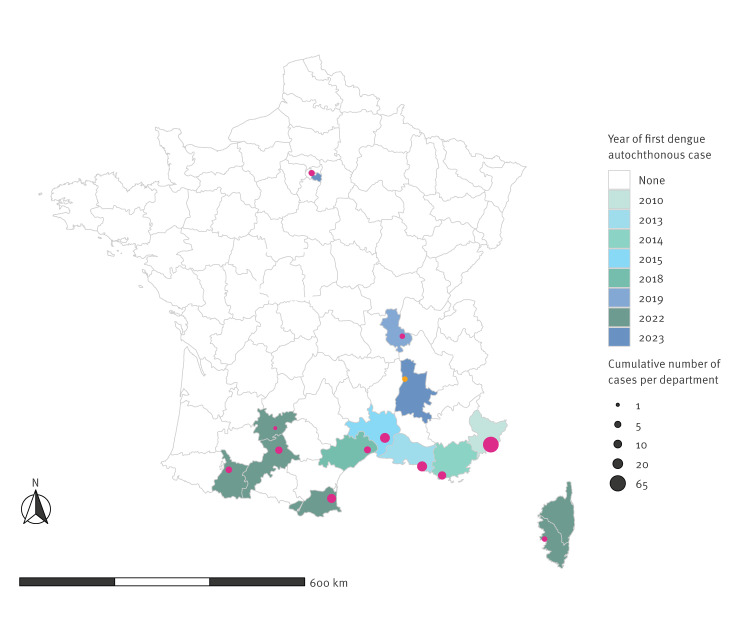
Reported autochthonous dengue cases per department in mainland France, 2010–2023 (n = 140 cases)

Source: Data from *Santé Publique France* (https://www.santepubliquefrance.fr/maladies-et-traumatismes/maladies-a-transmission-vectorielle/chikungunya/articles/donnees-en-france-metropolitaine).

In mainland France, autochthonous DENV infections were initially detected in the city of Nice and these remained restricted to the south of the country until 2018 [6,9]. Since then, the dengue epidemiological pattern has evolved. In 2022 alone, the total of 65 autochthonous cases identified exceeded the cumulated number of cases reported in the 10 prior years. Furthermore, the latitudinal limit of dengue clusters observed in the country moved northward, first to the Auvergne-Rhône-Alpes region in 2019 and then to the Île-de-France region in 2023 [10,11]. For each department in mainland France, the year of first autochthonous dengue detection and the cumulated number of cases (up to 2023) are presented in Figure 1.

Fourteen departments reported autochthonous dengue since the first detection in 2010 in the Alpes-Maritimes department. The cumulated number of dengue cases per department since the first detection is indicated (purple dots). The location of the current cluster is indicated (orange dot).

All dengue clusters reported in mainland France have occurred in areas colonised by the Asian tiger mosquito, *Aedes albopictus*, which up to December 2023 was detected in 75 of 96 departments [12].

In Europe *Ae. albopictus* is generally considered as the vector of dengue virus (DENV) due to several criteria that this mosquito species fulfils: *Ae. albopictus* colonises regions where autochthonous transmission events have occurred, is highly anthropophilic and transmits DENV in laboratory vector competence assays [13,14]. Another additional and important criterion to confirm a species as a vector is the detection of DENV-infected field specimens. As the rate of DENV infection in mosquitoes might be low [15], finding such specimens may be challenging. So far, only two studies have reported DENV-positive field-collected *Ae. albopictus* in Europe [16,17], and these studies were respectively in Spain (2015) and Italy (2023), two countries that conduct entomo−virological monitoring upon detection of dengue cases [16,17].

In mainland France, entomological surveillance is not constitutively implemented by health authorities around autochthonous cases. The reasons for this are that collecting and analysing mosquitoes is time-consuming, labour-intensive and can potentially interfere with outbreak management when human sampling and mosquito control remain priorities. Nevertheless, having firm evidence that a mosquito species is a DENV vector in a country may stimulate research on this species (e.g. to investigate its transmission potential or its insecticide-resistance patterns). Moreover, DENV-infected mosquitoes retrieved near human clusters can serve as a source of virological material for epidemiological surveillance. This can be especially helpful when human samples are lacking (e.g. no detectable or low viraemia, case absent during visitations or case not wishing to be sampled).

In this context, a network was created in France in 2022 between the *Agence Régionale de Santé* (ARS) en Auvergne-Rhône-Alpes (Regional Health Agency of Auvergne-Rhône-Alpes; ARS ARA), *Santé Publique France* (French national public health agency; SPF), the *Entente Interdépartementale Rhône-Alpes pour la Démoustication* (Rhône-Alpes inter-departmental agreement for mosquito control; EIRAD), the *Centre National de Référence des arbovirus* (National Reference Centre for arboviruses; CNR) and the UMR754 *Infections Virales et Pathologie Comparée* (joint research unit 754 for viral infections and comparative pathology; IVPC) to support public policies towards mosquito-borne diseases.

### Outbreak detection

In late summer 2023, two individuals with a clinical presentation compatible with dengue were identified through the national surveillance system in an urban area situated in Auvergne-Rhône-Alpes, France. As they had not travelled to an area with ongoing dengue transmission in the 2 weeks before their symptom onset, they were suspected of being autochthonous. Laboratory tests confirmed that they had been infected by DENV and because *Ae. albopictus* was known to be present in their neighbourhood, further epidemiological investigations were conducted, including searching for additional cases. These investigations were coupled with a novel entomo−virological surveillance approach that we tested. The approach consisted in collecting mosquitoes from private traps of citizens living within the human cluster area, as well as identifying the trapped mosquitoes’ species, and attempting to find a mosquito-borne virus for further virus tracing.

Here, we describe the autochthonous dengue cluster, the epidemiological, entomological and virological investigations related to it, their findings, as well as the control measures that were applied to stop further spread of the virus.

## Methods

### Epidemiological investigations

While dengue is a mandatorily notifiable disease throughout the year in France since 2006, prevention work and surveillance of human cases are reinforced in mainland France during the activity period of *Ae. albopictus* (May to November). Diagnosis of dengue by health professionals is notified to the ARS, which, together with SPF, coordinates an outbreak response and epidemiological investigations around each dengue case, further defined as follows.

#### Case definition

A suspected case is defined as an individual with a sudden high-grade fever (> 38.5 °C) associated with one of the following clinical signs (headache, myalgia, arthralgia, lower back pain, retro-orbital pain) which cannot be explained by another medical condition. A probable case is defined as a suspected case with a positive serology for immunoglobulin (Ig)M antibodies to *Orthoflaviviruses*. A confirmed case is defined as a suspected case with positive laboratory tests (real-time RT-PCR or positive serology for IgM and IgG antibodies to DENV). Autochthonous or imported refers to the travel history of the case. In absence of travel history within 2 weeks prior to symptoms onset, the case is considered autochthonous.

#### Active case finding

Upon autochthonous case detection, an active case finding is implemented within a 200-m radius around the cases that corresponds to the estimated flight range of *Ae. albopictus* mosquitoes [18]. A door-to-door epidemiological survey is implemented within this area to determine the extent of virus circulation with the use of fingertip blood tests for suspected cases.

#### Laboratory methods

The CNR is responsible for laboratory confirmation of the cases using a microneutralisation assay and/or reverse-transcription quantitative PCR (RT-qPCR) depending on the day of symptom onset (DSO). In this study, serum samples (prediluted 1/20) were serially diluted 12 times (twofold dilutions). The contact step, which allows specific antibodies to bind to infectious virus particles and subsequently inhibit virus growth, was performed in a 220 μL volume for 1 hour at 37 °C using dengue virus serotypes 1, 2, 3, 4 and West Nile virus (WNV) (source: CNR). This corresponded to 0.5 tissue culture infectious dose (TCID50) per μL of final serum dilution. A total of 100 μL of this mixture was inoculated onto Vero E6 cells in a 96-well microplate format. The test was automated in a biosafety level (BSL3) laboratory for all dilution and dispensing steps and for cytopathic effect (CPE) reading (the presence or absence of a CPE was evaluated). The test was performed in a duplicate format. Neutralisation titres > 20 were considered positive. Virus RNA sequencing and/or virus isolation is performed if the viral load (as estimated by the quantification cycle, Cq) is sufficient, generally Cq < 35 [19]. Other arboviruses such as Zika virus (ZIKV), chikungunya virus (CHIKV) and WNV are systematically tested.

### Entomological investigations

Upon detection of a suspected case, the colonisation status of *Ae. albopictus* in the places where the concerned individual has been/is while viraemic is verified online on the *système d'information du ministère chargé de la santé dédié à la prévention des maladies vectorielles* (Information system of the Ministry of Health dedicated to the prevention of vector-borne diseases; SI-LAV). Field investigations are performed by vector control operators (EIRAD) within a 200-m radius around the places to verify the presence of the Asian tiger mosquito (main potential vector). Larvae and adult mosquitoes are collected both in public and private areas (upon acceptance) then identified at the species level using dichotomous keys. The first confirmed observation of an *Ae. albopictus* specimen (either larvae or adult) stops the investigations and the area is considered colonised. During entomological investigations, operators perform manual removal of small size larval breeding sites (i.e. emptying small recipients with standing water) and process to larvicide application (*Bacillus thuringiensis* var. *israelensis* and *Bacillus sphericus* microgranules, VectoMax formulation, approx. 10 g/50 L) in inaccessible breeding sites (e.g. rainwater downspout). 

Upon case confirmation, ARS and SPF initiate vector control operations that target adult *Ae. albopictus* mosquitoes in the 200-m radius using Deltamethrin (see Outbreak control measures section). 

When residences are present in the 200-m radius, members of EIRAD attempt to contact their inhabitants concerning mosquito control activities and get their consent to conduct these on private properties. We proposed to add, during home’s visitation by vector control operators, the collection (with permission of the house owners) of any adult mosquitoes present in private individual mosquito traps. Upon collection, mosquitoes were stored at − 20 °C for 3 days then transferred to partners with suitable facilities for mosquito and arbovirus identification, who processed the samples immediately. Soft forceps were used to avoid damaging specimens. Each individual mosquito was observed under a binocular (5× magnification) and sorted by species. Particular attention was paid to avoid the presence on each specimen of mosquito body parts (such as a leg) from another individual.

### Detection of dengue virus RNA in mosquitoes in the radius around autochthonous cases

Mosquitoes were homogenised at IVPC under BSL 3 conditions in Leibovitz’s L-15 culture media using a Qiagen TissueLyser II apparatus. Total RNA was isolated from 100 µL of lysate using Macherey–Nagel NucleoSpin 96 virus kit according to the manufacturer’s recommendations. Presence of arbovirus genetic material (i.e. RNA) in mosquito pools was tested at the *Agence Nationale de Sécurité sanitaire de l’alimentation, de l’environnement et du travail* (French Agency for Food, Environmental and Occupational Health and Safety; ANSES) using a previously validated Real-Time RT-qPCR TaqMan BioMark assay (hereafter designed as TaqMan BioMark assay) targeting 64 arboviruses that notably allows a serotype-specific detection for each of the four DENV serotypes [20]. Actin amplification served as control for nucleic acid extraction and spiked *Escherichia coli* DNA amplification served as control for presence of potential PCR inhibitors.

Genetic material from the DENV of serotype 2 (DENV-2) positive mosquito pool found in the context of the current dengue cluster investigation was sent to the CNR for sequencing and phylogenetic analysis.

### Sequencing and phylogenetic analysis of dengue virus

Amplicon-based virus sequencing allowed us to obtain 1,929 nt encompassing part of the prM (290 nt), the full-length envelope (1,485 nt), and part of the non-structural protein 1 (NS1; 154 nt) coding sequences. This partial sequence from the DENV-2 positive *Ae. albopictus* mosquito pool was analysed in combination with: (i) all nearly complete available genomes (> 8,500 nt) from the Cosmopolitan genotype with sampling date and location information (as at November 2023), and (ii) a set of sequences from the 2023–2024 epidemic in the French Caribbean Islands [21]. The accession numbers of the sequences used for this analysis are detailed in the Supplementary Table 1. Phylogenetic relationships were inferred using ModelFinder and a Maximum-Likelihood approach implemented within IQTREE (v1.6.12) with ultrafast bootstrap approximation (1,000 replicates) [22,23].

## Results

### Epidemiological findings

In late summer 2023, through the national surveillance system, the ARS ARA was notified of two suspected dengue cases (Cases 1 and 2) in a town in Auvergne-Rhône-Alpes (Figure 1, orange dot) [24]. Cases 1 and 2 lived in the same house, in an urban residential area and presented typical dengue clinical symptoms with high-grade fever, headache and myalgia; Case 1 additionally had maculopapular rash. The day of symptom onset (DSO) for Case 1 was considered here as a reference (i.e. day 1). Accordingly, Case 2 DSO was day 14. Epidemiological investigations led by the ARS ARA and SPF confirmed, for Cases 1 and 2, the absence of travel in an area with ongoing dengue transmission within a 2-week period before DSO. This led to their classification as suspected autochthonous cases. During epidemiological investigations, active case finding identified an individual (Case 3) in the neighbourhood of the two cases, who 22 days before the DSO of Case 1, had returned from a French overseas territory in the Caribbean with an ongoing dengue epidemic [21]. Case 3 exhibited typical dengue symptoms 2 days after arrival from the Caribbean Islands but, at that time, the medical teleconsultation did not result in a dengue diagnosis. Suspected imported Case 3 DSO occurred 21 days prior Case 1.

Serum samples were submitted to the CNR, which confirmed DENV infections in Cases 1 and 2, based on the detection of DENV-specific antibodies using a combination of enzyme-linked immunosorbent (ELISA) and microneutralisation assays for Case 1, and based on a positive RT-qPCR for Case 2. For the latter case, virus isolation and sequencing were attempted but failed, likely due to the low viral load in the sample (Cq > 35). Case 3 was reported as a probable imported DENV infection based on the detection of anti-flavivirus IgMs (ELISA).

Door-to-door case finding was conducted within a 200-m radius from the confirmed cases, targeting 171 houses, of which 116 (68%) could be examined and resulting in four more suspected cases. For the latter, blood tests returned negative for DENV after analysis by the CNR or local laboratory.

**Figure 2:**
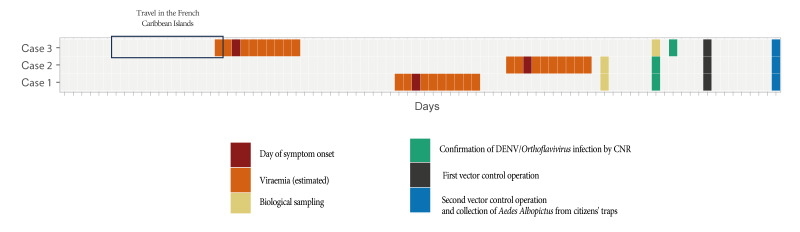
Timeline of events in an autochthonous human dengue cluster in a town of Auvergne-Rhône-Alpes, France, late summer 2023 (n = 3 cases)

CNR: National Reference Centre; DENV: dengue virus.

The events’ timeline is summarised in Figure 2. Cases 1 and 2 only reported one travel outside the 200-m radius during their estimated viraemia but the sites they had visited were not suitable for *Ae. albopictus* (mineral zone, elevated floor), no bite was reported, and the time spent at the sites was short. Therefore, these sites were not considered for epidemiological nor entomological investigations.

Timeline of events based on the epidemiological investigations (each square represents a day).

### Entomological findings

**Figure 3:**
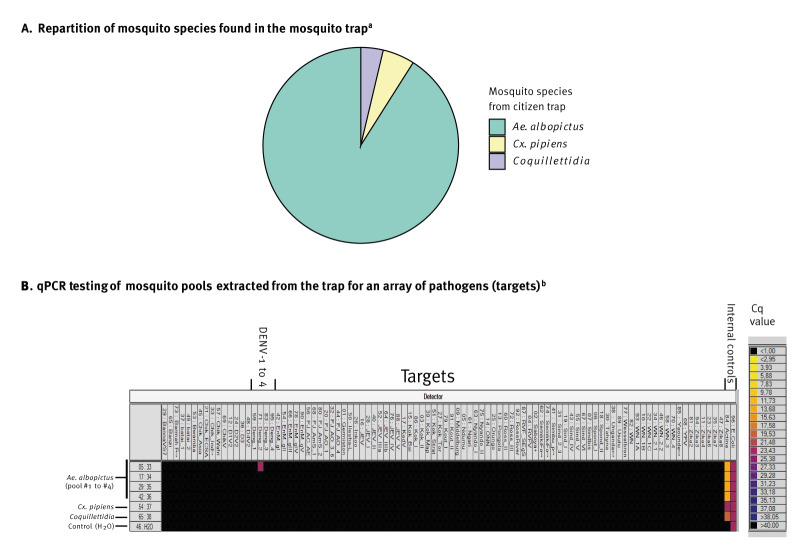
Detection of RNA from dengue virus serotype 2 in *Aedes albopictus* mosquitoes, caught in a private trap in an area concurrently affected by a cluster of dengue cases after (A) separating mosquito species and (B) testing pools for viral infection, Auvergne-Rhône-Alpes, France, 2023 (n = 4 mosquito pools)

The presence of *Ae. albopictus*, was previously reported in the town where the cases resided (SI-LAV, 2017). In the cluster area, operators had access to 55 of the 188 houses (29%) targeted during the vector control operations. A single trap (BG-Mosquitaire, Biogents) was identified in a house visited prior the second treatment. This house was in the vicinity of the garden of Case 3, although separated by a large grove of trees. Owners declared that their trap had been running uninterruptedly since the beginning of the summer and they accepted to give its content to EIRAD investigators. Collected specimens were identified at the species or genus level using determination keys [25]. There were in total 132 individual mosquitoes, of which 120 belonged to the *Ae. albopictus* (91%) species, seven to the *Culex pipiens* (5.3%) species and five to the *Coquilletidia* genus (3.7%) (Figure 3A). The collected mosquitoes were relatively dry, suggesting relatively old specimens, although the exact capture date could not be determined. Following identification at the species/genus level, mosquitoes were pooled by species/genus with four pools of 30 *Ae. albopictus*, one pool of *Cx. pipiens* and one pool of *Coquilletidia*. Specimens were immediately processed for DENV RNA detection.

*Ae. Albopictus*: *Aedes albopictus*; Cq: quantification cycle; *Cx pipiens*: *Culex pipiens*; qPCR: quantitative PCR.

^a^ The trap was in a private home in an urban neighbourhood concurrently affected by dengue autochthonous cases; mosquitoes were sampled from it in late summer 2023.

^b^ The output of the high throughput qPCR TaqMan BioMark assay shows the detection of DENV-2 RNA from one of the *Ae. albopictus* pools. Other targets correspond to other mosquito-borne viruses as previously described in [20]. The Cq of around 25 for the DENV-2-positive *Ae. albopictus* pool derived from the high throughput assay is lower than the of Cq of 33.8 found by a qPCR in the National Reference Centre for the same pool. This is because of the high-throughput qPCR is conducted after a step of virus RNA pre-amplification.

### Virological findings

**Figure 4:**
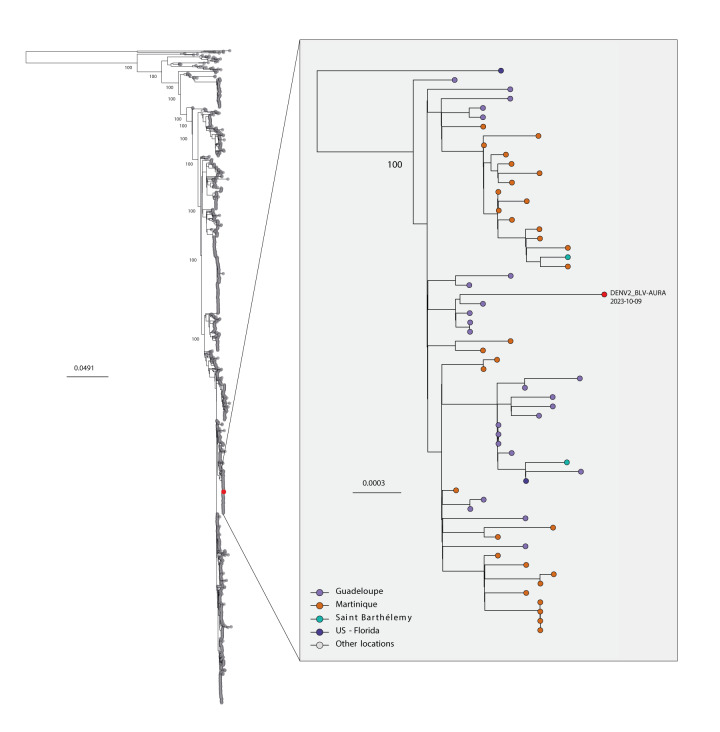
Phylogenetic relations between the dengue virus serotype 2 detected in mosquitoes, which were trapped in an area concurrently affected by a cluster of dengue cases in Auvergne-Rhône-Alpes and other dengue viruses from the dengue epidemic in the French Caribbean Islands, 2023–2024

DENV-2: dengue virus serotype 2; US: United States.

Beta-actin was detected at low Cq (from 10 to 25) in the six mosquito pools tested. One pool of *Ae. albopictus* was positive in TaqMan BioMark assay for DENV-2. This was confirmed by PCR amplification performed by the CNR on a second sample from the same pool (Cq value of 33.8). No other arbovirus RNA was detected by TaqMan BioMark assay in any of the samples tested (Figure 3B). Therefore, the minimum prevalence of DENV infection in *Ae. albopictus* mosquitoes is 0.83% (i.e. one infected specimen out of 120). Sequencing and analysis of the partial virus genome showed that the virus strain isolated from mosquitoes (DENV-2_BLV-AURA) belonged to the Cosmopolitan genotype of DENV-2. Phylogenetic analysis of the partial sequence combined with sequences from the Cosmopolitan genotype and recent sequences from the French Caribbean islands showed that the DENV-2_BLV-AURA strain derived from a lineage from the French Caribbean Islands (Figure 4).

Numbers on deeper nodes of the trees represent bootstrap values. The scale bars are proportional to the number of substitutions per site along the branches of the respective trees. The accession numbers of the sequences used for this analysis are detailed in Supplementary Table 1. Tips are coloured according to the sampling location (or country of infection for imported cases). The virus detected in the current study (DENV2-BLV-AURA 2023-10-09) is specified as belonging to DENV-2, Cosmopolitan genotype. A.

### Outbreak control measures

A press statement (https://www.auvergne-rhone-alpes.ars.sante.fr/suspicion-de-deux-cas-autochtones-de-dengue-dans-la-drome-26) was published 2 days after the detection of Cases 1 and 2 to provide essential information about the ongoing dengue cluster at the time, as well as recommendations to limit mosquito bites (individual protection and control measures). Active case finding by door-to-door investigation (see results) and the removal of mosquito breeding sites in the visited homes during entomological investigations (see methods) limited the spread of the cluster.

Vector control operations targeting adult *Ae. albopictus* mosquitoes consisted of two successive operations, performed 1 week apart. The first treatment consisted of an ultra-low volume pulverisation, while walking, of deltamethrin (by cold nebulisation, 1 g/hectare) on the vegetation (mosquito resting area) of all private homes that allowed access, combined with an ultra-low volume spraying of deltamethrin (by cold nebulisation, 1 g/hectare) from a pick-up car on the roadway to target the vegetation in the public space. This was done in the whole 200-m radius around the confirmed cases’ houses. The second treatment, 1 week later, was similar to the first except that spraying of deltamethrin in private gardens was performed only in the homes close (e.g. within the same dead-end) to the confirmed cases (representing 17 residences from which 14 accepted the treatment). Advertising for vector control operations was made 48 h in advance by distributing flyers that summarised general information such as the date, time of treatment and safety instructions to limit human exposure to adulticide (avoid being present, remove objects from the garden and avoid consumption of garden products for a week). All the treatments were performed at night. Another larvicidal treatment could be performed in the private houses upon advertising for the second adulticide treatment, if needed.

## Discussion

This study provides the proof-of-concept that coupling epidemiological investigations with entomo−virological surveillance based on mosquitoes collected from existing peri-domestic private traps within an autochthonous cluster area can be an effective tool to track arbovirus circulation.

Taken together, our epidemiological and entomo-virological data strongly support a link between the probable index case (Case 3) and the two autochthonous cases in Auvergne-Rhône-Alpes, and a link between this cluster and the epidemic in the French Caribbean Islands [21].

Case 3 was likely misdiagnosed during teleconsultation, showing the limits of this type of consultation and the importance of the regular communications by ARS and SPF to bring mosquito-borne diseases to the attention of healthcare professionals in mainland France.

The mosquitoes collected in this study are the first DENV-infected field specimens identified in mainland France, despite previous collections and detection attempts around autochthonous dengue clusters in the south of France (Albin Fontaine, personal communication, March 2024). Prior to the dengue cluster in Auvergne-Rhône-Alpes in 2023, field-collected mosquitoes infected by DENV were detected only twice in Europe in Spain (2015) and Italy (2023), both times in pools of *Ae. albopictus* [16,17]. Together, this body of evidence supports the role of *Ae. albopictus* as a vector during autochthonous DENV transmission in Europe.

The estimated cases’ viraemia (set at − 2 and + 7 DSO based on CNR recommendations according to literature data [26]) suggests that *Ae. albopictus* was exposed to DENV between 22 days (at the earliest, with Case 2) and 56 days (at the latest, with Case 3) before collection, assuming there were no unidentified cases in the area. Based on semi-field experiments, some *Ae. albopictus* individuals can survive up to 45 days upon optimal conditions, even though only 10% of the population survives for at least 20 days [27]. It is generally considered that virus infection has a negligible effect on mosquito lifespan [28]. Therefore, it is likely that several independent mosquito infection events occurred although we cannot exclude that a single female was responsible for all DENV transmission in the cluster.

Current knowledge on European *Ae. albopictus* transmission potential for DENV remains scarce. Laboratory studies on vector competence (i.e. the ability of a vector to become infected then subsequently retransmit the virus) indicate that at least 7 days are required after an infectious blood meal to detect infectious particles in the mosquito’s saliva, with only up to ca 40% of mosquitoes being infectious at day 21 post infectious blood meal depending on the study [14,29-31]. Parameters that influence vector competence include, but are not limited to, mosquito genotype, mosquito microbiome, virus genotype, virus dose (as a proxy for human viraemia) under the influence of environmental factors such as temperature [32]. More data are needed on *Ae. albopictus* transmission potential for a panel of European mosquito and virus strains. Notably, this would consider intra-vector infection dynamics which, combined to epidemiological modelling would help to better understand, anticipate and predict DENV circulation in Europe [33,34]. Beyond vector competence of a mosquito species, the potential of DENV transmission among humans also depends on vector-related (density per host, daily survival and daily biting rate per human) as well as host-related traits as proposed in the vectorial capacity model [35]. Data related to these traits, some of which must be measured in the field, are often lacking to accurately estimate the full transmission potential of a mosquito–arbovirus pair. Altogether, this partly explains why no association was found between the number of travel-related cases and occurrence of autochthonous outbreaks in Europe and advocates for further studies on mosquito vectorial capacity for arboviruses in Europe [36].

Surveillance of mosquitoes and arboviruses is key for control management as it facilitates identification of vector species and pathogen tracing. Notably, through active surveillance around declared human cases, circulating viruses can be isolated. While passive surveillance of mosquitoes in absence of declared human cases is useless for non-endemic viruses like DENV, it can improve early warning for endemic arboviruses such as WNV [37]. Recently developed tools such as MX trap adapter for collection of mosquito excreta or high-throughput microfluidic PCR array have proven their efficacy and these could contribute to a renewal of surveillance methods [20,38].

The number of private adult mosquito traps is increasing in mainland France, mostly due to *Ae. albopictus* nuisance, making them a valuable source of biological information for outbreak investigations notably when human samples from which virological material can be retrieved are lacking. Collection of mosquitoes through the traps has limited impact on outbreak management operations as this happens during mandatory homes’ visits before vector control, is quick to implement and does not require any specific material. While mosquito collection from private traps will likely not be possible upon every dengue autochthonous case, we argue that it should be routinely attempted given its simplicity, rapidity and potential cost-effectiveness. In Auvergne-Rhône-Alpes, in the case of an autochthonous cluster where the use of adulticides is not possible (mainly due to ecotoxological risk), the protocol in place includes the deployment of adult traps around the cluster area. The collected mosquitoes, which are sorted and numerated by species to estimate the effectiveness of this alternative mosquito-control approach could also be used to obtain virological information.

A recent study showed that DENV RNA could be detected in 50% of infected mosquito specimens for up to 8 weeks when incubated at 21–22 °C [39]. Here, DENV RNA was isolated and partially sequenced from dead, dry mosquito specimens that spent at least 20 days in a trap experiencing the summer temperatures (likely 25–30 °C) of the locality in question. Altogether, this underlines the feasibility of this procedure that could be implemented as a routine during autochthonous case management in France and in Europe.

Identifying and confirming that a species acts as a vector motivates operational research on this species, as exemplified by the recent description of the genetic insecticide resistance pattern of *Ae. albopictus* populations from 95 sites along the south coast of France [40]. In addition, the confirmation of the vector species through the detection of field-infected vectors guides public policies against vector-borne diseases. It can act as a powerful driver for community engagement and could be used as a lever towards better vector management [41].

Raising citizen awareness about the value of the content of their mosquito traps upon dengue case detection could facilitate surveillance and epidemiological monitoring. More broadly, citizen effort for mosquito surveillance for instance through citizen science projects has proven is worth, notably for mosquito species monitoring (including recently introduced invasive species) as shown in several European countries [42,43]. This could also represent an interesting tool for arbovirus surveillance at larger scales, in complement to expert surveillance, for pathogens with an endemic circulation such as WNV or Usutu virus in Europe [44].

Since the first detection of autochthonous dengue in France in 2010, no additional cases were detected around any investigated cluster pointing to the effectiveness of the current control measures. However, citizen acceptance of outbreak response procedures (visits, biological tests, vector control) often remains incomplete. As one limitation in our study, only 29% of homes targeted during the first vector control operation accepted this measure; moreover 14 of 17 homes targeted during the second vector control operation accepted this. We could have therefore missed mosquitoes of interest for our investigation. Another limitation is that we analysed mosquitoes in pools to test for DENV infection, preventing retrieval of information at individual mosquito level.

Anthropological studies could help to identify factors (e.g. risk perception) that could influence the levels of citizen engagement towards vector-borne diseases management from vector control acceptance to participation in the surveillance effort. Entomo−virological surveillance could also be improved by processing collected mosquitoes individually (instead of pools) and at the tissue level (instead of entire specimens) in order to better estimate the proportion of infected mosquitoes and if they present a disseminated infection, which is a proxy of their transmission potential.

### Conclusion

This study reminds the importance of raising mosquito-borne disease awareness among healthcare professionals and the general public and provides further evidence that *Ae. albopictus* is a DENV vector in mainland France. This work highlights the benefits of entomo−virological surveillance for public health and supports its implementation in the current procedures. It illustrates how networks gathering public health stakeholders, researchers and citizens form a powerful resource to improve the management of mosquito-borne diseases, in complement to current measures in place.

## Supplementary Data

233000 Supplementary Table 1
