# Supplementary material for: Peri-domestic entomological surveillance using private traps allows detection of dengue virus in Aedes albopictus during an autochthonous transmission event in mainland France, late summer 2023
Source: Euro Surveill. 2024 Sep 5;29(36):2400195. doi: 10.2807/1560-7917.ES.2024.29.36.2400195 (PMC11378516; doi:10.2807/1560-7917.ES.2024.29.36.2400195)
Supplement: Supplementary Table 1 [file 2400195_SupplementaryTable1.pdf]

This supplementary material is hosted by *Eurosurveillance* as supporting information alongside the article **Peri-domestic entomological surveillance using private traps allows detection of dengue virus in *Aedes albopictus* during an autochthonous transmission event in mainland France, late summer 2023**, on behalf of the authors, who remain responsible for the accuracy and appropriateness of the content. The same standards for ethics, copyright, attributions and permissions as for the article apply. Supplements are not edited by *Eurosurveillance* and the journal is not responsible for the maintenance of any links or email addresses provided therein.

Supplementary table 1. Names and Genbank accession numbers of the dengue virus RNA partial and complete sequences used for phylodynamic analysis in the study. See also Klitting *et al.*, Eurosurveillance, 2024 (https://doi.org/10.2807/1560-7917.ES.2024.29.13.2400123).

SequenceID	Genbank_accesssion
DENV2_BLV-AURA_2023-10-09	PP326834
65364|DENV2|MTQ|2023-2-18	PP326744
65244|DENV2|MTQ|2023-2-14	PP326743
65877|DENV2|MTQ|2023-4-19	PP326746
66202|DENV2|MTQ|2023-5-11	PP326747
66353|DENV2|GLP|2023-4-4	PP326748
66354|DENV2|MTQ|2023-4-18	PP326749
66356|DENV2|GLP|2023-4-24	PP326750
66357|DENV2|MTQ|2023-4-25	PP326751
66358|DENV2|GLP|2023-4-25	PP326752
66362|DENV2|MTQ|2023-5-12	PP335483
66364|DENV2|GLP|2023-5-19	PP326755
66366|DENV2|GLP|2023-5-20	PP326757
66367|DENV2|MTQ|2023-5-19	PP326758
66368|DENV2|GLP|2023-5-23	PP326759
66475|DENV2|GLP|2023-6-6	PP326760
66482|DENV2|GLP|2023-6-6	PP326761
66483|DENV2|MTQ|2023-6-7	PP326762
66484|DENV2|MTQ|2023-6-6	PP326763
66485|DENV2|MTQ|2023-6-7	PP326764
66593|DENV2|MTQ|2023-6-9	PP326765
66594|DENV2|MTQ|2023-6-13	PP326766
66595|DENV2|GLP|2023-6-12	PP326767
66596|DENV2|GLP|2023-6-12	PP326768
66597|DENV2|GLP|2023-6-13	PP326769
66897|DENV2|GLP|2023-6-21	PP326770
66898|DENV2|GLP|2023-6-22	PP326771
66901|DENV2|MTQ|2023-6-27	PP326772
66902|DENV2|GLP|2023-6-26	PP326773
66904|DENV2|MTQ|2023-6-27	PP326774
66905|DENV2|MTQ|2023-6-27	PP326775
66906|DENV2|MTQ|2023-6-27	PP331235
66909|DENV2|GLP|2023-6-29	PP326776
67008|DENV2|GLP|2023-7-3	PP326777
67187|DENV2|MTQ|2023-7-3	PP326778
67188|DENV2|MTQ|2023-7-3	PP326779
67189|DENV2|MTQ|2023-7-5	PP326780

67190|DENV2|MTQ|2023-7-5	PP326781
67192|DENV2|GLP|2023-7-6	PP326782
67198|DENV2|MTQ|2023-7-7	PP326783
67201|DENV2|MTQ|2023-7-11	PP326784
67561|DENV2|MTQ|2023-7-26	PP326785
67618|DENV2|MTQ|2023-8-2	PP326786
68638|DENV2|GLP|2023-08-19	PP326790
68641|DENV2|GLP|2023-8-17	PP326791
68649|DENV2|MTQ|2023-8-17	PP326793
68925|DENV2|MTQ|2023-08-23	PP326797
69212|DENV2|GLP|2023-08-29	PP326800
69709|DENV2|GLP|2023-09-05	PP326803
70053|DENV2|MTQ|2023-09-26	PP326813
70621|DENV2|GLP|2023-10-12	PP326814
70694|DENV2|GLP|2023-08-29	PP326816
71024|DENV2|BLM|2023-10-06	PP326820
71027|DENV2|BLM|2023-10-05	PP326821
71050|DENV2|MTQ|2023-08-07	PP326822
71056|DENV2|MTQ|2023-09-12	PP326823
71057|DENV2|MTQ|2023-08-24	PP326824
71333|DENV2|GLP|2023-11-01	PP326825
AB122020|III-S-Asian-America|Dominican-Republic|2001	AB122020
AB189122|Indonesia|Unk	AB189122
AB189123|Indonesia|Unk	AB189123
AB189124|Indonesia|Unk	AB189124
AF038403|IV-Asian-II|Unknown|Unknown	AF038403
AF100469|I-American|Unknown|Unknown	AF100469
AF204177|IV-Asian-II|China|Unknown	AF204177
AF489932|III-S-Asian-America|Unknown|Unknown	AF489932
AY037116|Australia|Unk	AY037116
AY702036|III-S-Asian-America|Cuba|1997	AY702036
AY776328|Taiwan|Unk	AY776328
AY858035|Indonesia|Unk	AY858035
AY858036|Indonesia|Unk	AY858036
DQ181797|V--Asian-I|Thailand|2001	DQ181797
DQ181798|V--Asian-I|Thailand|1999	DQ181798
DQ181802|V--Asian-I|Thailand|1988	DQ181802
DQ181804|V--Asian-I|Thailand|1984	DQ181804
DQ181805|V--Asian-I|Thailand|1979	DQ181805
DQ181806|V--Asian-I|Thailand|1974	DQ181806
DQ645540|Taiwan|Unk	DQ645540
DQ645541|Taiwan|Unk	DQ645541
DQ645542|Taiwan|Unk	DQ645542
DQ645543|Taiwan|Unk	DQ645543
DQ645544|Taiwan|Unk	DQ645544
DQ645545|Taiwan|Unk	DQ645545
DQ645546|Taiwan|Unk	DQ645546
DQ645547|Taiwan|Unk	DQ645547
DQ645548|Taiwan|Unk	DQ645548
DQ645549|Taiwan|Unk	DQ645549
DQ645550|Taiwan|Unk	DQ645550
DQ645551|Taiwan|Unk	DQ645551

DQ645552|Taiwan|Unk	DQ645552
DQ645553|Taiwan|Unk	DQ645553
DQ645554|Taiwan|Unk	DQ645554
DQ645555|Taiwan|Unk	DQ645555
DQ645556|Taiwan|Unk	DQ645556
EF051521|China|2001	EF051521
EF105379|VI-Sylvatic|Malaysia|1970	EF105379
EF105382|VI-Sylvatic|Burkina-Faso|1980	EF105382
EF105387|VI-Sylvatic|Nigeria|1966	EF105387
EF105389|VI-Sylvatic|Senegal|1999	EF105389
EU056810|Burkina-Faso|1983	EU056810
EU056811|I-American|Peru|1995	EU056811
EU056812|I-American|Puerto-Rico|1977	EU056812
EU081177|II-Cosmopolitan|Singapore|2005	EU081177
EU081178|Singapore|2005	EU081178
EU081179|II-Cosmopolitan|Singapore|2005	EU081179
EU081180|II-Cosmopolitan|Singapore|2005	EU081180
EU179857|Brunei|2005	EU179857
EU179858|Brunei|2005	EU179858
EU179859|II-Cosmopolitan|Brunei|2006	EU179859
EU359009|China|Unk	EU359009
EU482582|III-S-Asian-America|USA|1989	EU482582
EU482640|Viet-Nam|2006	EU482640
EU687216|III-S-Asian-America|USA|2005	EU687216
EU687217|III-S-Asian-America|USA|2005	EU687217
FJ196853|China|2003	FJ196853
FJ467493|VI-Sylvatic|Malaysia|2008	FJ467493
FJ898461|III-S-Asian-America|Belize|2002	FJ898461
FM210211|V--Asian-I|Viet-Nam|2003	FM210211
GQ199868|III-S-Asian-America|Nicaragua|2007	GQ199868
GQ398258|Indonesia|1975	GQ398258
GQ398259|Indonesia|1976	GQ398259
GQ398260|Indonesia|1976	GQ398260
GQ398261|Indonesia|1976	GQ398261
GQ398262|Indonesia|1976	GQ398262
GQ398263|Indonesia|1975	GQ398263
GQ398264|Indonesia|1976	GQ398264
GQ398265|Singapore|2008	GQ398265
GQ398266|Singapore|2007	GQ398266
GQ398267|Singapore|2007	GQ398267
GQ398290|III-S-Asian-America|Puerto-Rico|1994	GQ398290
GQ868540|III-S-Asian-America|Venezuela|1990	GQ868540
GQ868545|V--Asian-I|Unknown|1996	GQ868545
GU131843|Burkina-Faso|1986	GU131843
GU131896|V--Asian-I|Cambodia|2007	GU131896
GU370050|Singapore|Apr-2007	GU370050
HM488257|Guam|2001	HM488257
HQ891023|IV-Asian-II|Taiwan|2008	HQ891023
HQ999999|III-S-Asian-America|Guatemala|2009	HQ999999
JF327392|Singapore|2009	JF327392
JN851113|Singapore|2006	JN851113
JN851114|Singapore|2007	JN851114
JN851115|Singapore|2007	JN851115

JN851116|Singapore|2007	JN851116
JN851117|Singapore|2007	JN851117
JN851118|Singapore|2008	JN851118
JN851119|Singapore|2008	JN851119
JN851120|Singapore|2007	JN851120
JN851121|Singapore|2008	JN851121
JN851122|Singapore|2008	JN851122
JN851123|Singapore|2004	JN851123
JN851124|Singapore|2005	JN851124
JN851125|Singapore|2005	JN851125
JN851126|Singapore|2005	JN851126
JN851127|Singapore|2004	JN851127
JN851128|Singapore|2006	JN851128
JN851129|Singapore|2006	JN851129
JN851130|Singapore|2005	JN851130
JN851131|Singapore|2005	JN851131
JX470186|China|2010	JX470186
KC131142|China|Sep-2012	KC131142
KC762655|Indonesia|27-Sep-2007	KC762655
KC762656|Indonesia|11-Dec-2007	KC762656
KC762657|Indonesia|23-Apr-2008	KC762657
KC762658|Indonesia|14-Aug-2007	KC762658
KC762659|Indonesia|15-Apr-2008	KC762659
KC762660|II-Cosmopolitan|Indonesia|2007	KC762660
KC762661|Indonesia|24-Sep-2007	KC762661
KC762662|Indonesia|21-Nov-2007	KC762662
KC762663|Indonesia|20-Feb-2008	KC762663
KC762664|Indonesia|28-Mar-2008	KC762664
KC762665|Indonesia|06-Jul-2007	KC762665
KC762666|Indonesia|15-Feb-2008	KC762666
KC762667|Indonesia|12-Apr-2008	KC762667
KC762668|Indonesia|11-Mar-2008	KC762668
KC762669|II-Cosmopolitan|Indonesia|2007	KC762669
KC762670|Indonesia|11-Dec-2007	KC762670
KC762671|Indonesia|19-Feb-2008	KC762671
KC762672|Indonesia|09-Apr-2008	KC762672
KC762673|Indonesia|11-Mar-2008	KC762673
KC762674|Indonesia|11-Mar-2008	KC762674
KC762675|Indonesia|08-Mar-2008	KC762675
KC762676|Indonesia|17-Jul-2007	KC762676
KC762677|Indonesia|15-Feb-2008	KC762677
KC762678|Indonesia|04-Mar-2010	KC762678
KC762679|Indonesia|29-Mar-2010	KC762679
KC762680|II-Cosmopolitan|Indonesia|2010	KC762680
KC964093|China|2001	KC964093
KC964094|China|1993	KC964094
KF744397|Philippines|2001	KF744397
KF744398|Philippines|2005	KF744398
KF744406|IV-Asian-II|Philippines|1995	KF744406
KF744407|IV-Asian-II|Philippines|1996	KF744407
KM279514|Singapore|2011	KM279514
KM279515|Singapore|2011	KM279515
KM279516|Singapore|2011	KM279516

KM279517|Singapore|2011	KM279517
KM279518|Singapore|2011	KM279518
KM279519|Singapore|2011	KM279519
KM279520|Singapore|2011	KM279520
KM279521|Singapore|2011	KM279521
KM279522|Singapore|2011	KM279522
KM279524|Singapore|2011	KM279524
KM279525|Singapore|2011	KM279525
KM279526|Singapore|2011	KM279526
KM279527|Singapore|2011	KM279527
KM279528|Singapore|2012	KM279528
KM279529|Singapore|2012	KM279529
KM279531|Singapore|2012	KM279531
KM279532|Singapore|2012	KM279532
KM279533|Singapore|2012	KM279533
KM279535|Singapore|2012	KM279535
KM279537|Singapore|2012	KM279537
KM279538|Singapore|2012	KM279538
KM279542|Singapore|2012	KM279542
KM279543|Singapore|2012	KM279543
KM279544|Singapore|2012	KM279544
KM279546|Singapore|2012	KM279546
KM279547|Singapore|2012	KM279547
KM279549|Singapore|2012	KM279549
KM279550|Singapore|2012	KM279550
KM279551|Singapore|2012	KM279551
KM279552|Singapore|2012	KM279552
KM279555|Singapore|2012	KM279555
KM279556|Singapore|2012	KM279556
KM279557|Singapore|2012	KM279557
KM279560|Singapore|2012	KM279560
KM279561|Singapore|2012	KM279561
KM279562|Singapore|2012	KM279562
KM279564|Singapore|2012	KM279564
KM279566|Singapore|2012	KM279566
KM279567|Singapore|2012	KM279567
KM279568|Singapore|2012	KM279568
KM279569|Singapore|2012	KM279569
KM279570|Singapore|2012	KM279570
KM279571|Singapore|2012	KM279571
KM279572|Singapore|2012	KM279572
KM279573|Singapore|2012	KM279573
KM279574|Singapore|2012	KM279574
KM279575|Singapore|2012	KM279575
KM279578|Singapore|2012	KM279578
KM279579|Singapore|2012	KM279579
KM279580|Singapore|2013	KM279580
KM279581|Singapore|2011	KM279581
KM279582|Singapore|2011	KM279582
KM279583|Singapore|2011	KM279583
KM279584|Singapore|2011	KM279584
KM279585|Singapore|2011	KM279585
KM279586|Singapore|2011	KM279586

KM279587|Singapore|2012	KM279587
KM279588|Singapore|2012	KM279588
KM279589|Singapore|2012	KM279589
KM279590|Singapore|2012	KM279590
KM279591|Singapore|2012	KM279591
KM279592|Singapore|2012	KM279592
KM279593|Singapore|2012	KM279593
KM279594|Singapore|2012	KM279594
KM279595|Singapore|2012	KM279595
KM279596|Singapore|2012	KM279596
KM279597|II-Cosmopolitan|Singapore|2012	KM279597
KM279598|Singapore|2012	KM279598
KM279600|Singapore|2012	KM279600
KM279601|Singapore|2012	KM279601
KM279602|Singapore|2008	KM279602
KM279603|Singapore|2007	KM279603
KM279604|Singapore|2008	KM279604
KM279605|Singapore|2009	KM279605
KM279606|Singapore|2010	KM279606
KM279607|Singapore|2010	KM279607
KM279608|Singapore|2010	KM279608
KM279609|Singapore|2009	KM279609
KM279610|Singapore|2010	KM279610
KP012546|China|2014	KP012546
KP723478|China|2014	KP723478
KP723479|China|2010	KP723479
KR779782|Singapore|2007	KR779782
KR779786|Singapore|2013	KR779786
KT187553|China|2014	KT187553
KT187554|China|2014	KT187554
KT187555|China|2014	KT187555
KT187556|China|2014	KT187556
KT187557|China|2014	KT187557
KT187558|China|2014	KT187558
KU365901|Taiwan|2015	KU365901
KU365902|Taiwan|2015	KU365902
KU365903|Taiwan|2015	KU365903
KU509268|Indonesia|2009	KU509268
KU509269|Philippines|2009	KU509269
KU509272|Thailand|2009	KU509272
KU509274|Philippines|2010	KU509274
KU509275|Philippines|2008	KU509275
KU509276|Philippines|2008	KU509276
KU509277|Philippines|2010	KU509277
KU517845|Papua-New-Guinea|23-Apr-2013	KU517845
KU517846|Indonesia|07-May-2014	KU517846
KU517847|Philippines|03-Aug-2015	KU517847
KU666944|Malaysia|Jan-2014	KU666944
KU666945|Malaysia|Jan-2014	KU666945
KU666946|Malaysia|Feb-2014	KU666946
KU666947|Malaysia|Feb-2014	KU666947
KU666948|Malaysia|Feb-2014	KU666948
KU666949|Malaysia|Jan-2014	KU666949

KU948303|Singapore|23-Feb-2016	KU948303
KX225485|China|2015	KX225485
KX225486|China|2015	KX225486
KX372564|Australia|2015	KX372564
KX380807|Singapore|2012	KX380807
KX380808|Singapore|2012	KX380808
KX380809|Singapore|2012	KX380809
KX380810|Singapore|2012	KX380810
KX380811|Singapore|2012	KX380811
KX380812|Singapore|2012	KX380812
KX380813|Singapore|2012	KX380813
KX380814|Singapore|2012	KX380814
KX380815|Singapore|2012	KX380815
KX380816|Singapore|2012	KX380816
KX380817|Singapore|2012	KX380817
KX380818|Singapore|2012	KX380818
KX380819|Singapore|2012	KX380819
KX380820|Singapore|2012	KX380820
KX380821|Singapore|2012	KX380821
KX380822|Singapore|2012	KX380822
KX380823|Singapore|2012	KX380823
KX380824|Singapore|2012	KX380824
KX380825|Singapore|2013	KX380825
KX380826|Singapore|2013	KX380826
KX380827|Singapore|2013	KX380827
KX380830|Singapore|2013	KX380830
KX380831|Singapore|2013	KX380831
KX380832|Singapore|2013	KX380832
KX380833|Singapore|2013	KX380833
KX380834|Singapore|2013	KX380834
KX380835|Singapore|2013	KX380835
KX380836|Singapore|2013	KX380836
KX380837|Singapore|2013	KX380837
KX380838|Singapore|2013	KX380838
KX452015|Malaysia|Jan-2014	KX452015
KX452016|Malaysia|Jan-2014	KX452016
KX452017|Malaysia|Jan-2014	KX452017
KX452018|Malaysia|Jan-2014	KX452018
KX452019|Malaysia|Jan-2014	KX452019
KX452020|Malaysia|Jan-2014	KX452020
KX452021|Malaysia|Jan-2014	KX452021
KX452022|Malaysia|Jan-2014	KX452022
KX452023|Malaysia|Jan-2014	KX452023
KX452024|Malaysia|Jan-2014	KX452024
KX452026|Malaysia|Jan-2014	KX452026
KX452027|Malaysia|Jan-2014	KX452027
KX452028|Malaysia|Jan-2014	KX452028
KX452029|Malaysia|Jan-2014	KX452029
KX452030|Malaysia|Jan-2014	KX452030
KX452032|Malaysia|Jan-2014	KX452032
KX452033|Malaysia|Jan-2014	KX452033
KX452034|Malaysia|Jan-2014	KX452034
KX452035|Malaysia|Jan-2014	KX452035

KX452036|Malaysia|Jan-2014	KX452036
KX452037|Malaysia|Jan-2014	KX452037
KX452038|Malaysia|Jan-2014	KX452038
KX452039|Malaysia|Jan-2014	KX452039
KX452040|Malaysia|Jan-2014	KX452040
KX452041|Malaysia|Jan-2014	KX452041
KX452042|Malaysia|Jan-2014	KX452042
KX452043|Malaysia|Jan-2014	KX452043
KX452044|Malaysia|Jan-2014	KX452044
KX452045|Malaysia|Jan-2014	KX452045
KX452046|Malaysia|Jan-2014	KX452046
KX452047|Malaysia|Jan-2014	KX452047
KX452048|Malaysia|Jan-2014	KX452048
KX452049|Malaysia|Jan-2014	KX452049
KX621245|China|2015	KX621245
KX621246|China|2015	KX621246
KX621247|China|2015	KX621247
KX621248|China|2015	KX621248
KX655786|China|2015	KX655786
KX655787|China|2015	KX655787
KX655788|China|2015	KX655788
KY627762|Burkina-Faso|05-Nov-2016	KY627762
KY627763|Burkina-Faso|21-Nov-2016	KY627763
KY794785|Papua-New-Guinea|11-May-2010	KY794785
KY882458|China|Unk	KY882458
KY921904|Singapore|Mar-2014	KY921904
KY921905|Singapore|Mar-2015	KY921905
LC111438|East-Timor|Unk	LC111438
LC410189|Thailand|2016-10	LC410189
LC410190|Thailand|2016-10	LC410190
LC410191|Thailand|2017-02	LC410191
LC436669|Bangladesh|2017-10	LC436669
LC436672|Bangladesh|2017-10	LC436672
LC436673|Bangladesh|2017-11	LC436673
LC436674|Bangladesh|2017-11	LC436674
LC436675|Bangladesh|2017-12	LC436675
LC666718|Ghana|2017	LC666718
LC666719|Ghana|2017	LC666719
M20558|III-S-Asian-America|Unknown|Unknown	M20558
MF004385|France|06-Sep-2014	MF004385
MF043956|China|2016	MF043956
MF314189|Singapore|2016	MF314189
MG189962|Tanzania|Jun-2014	MG189962
MH010629|China|Nov-2017	MH010629
MH048671|Malaysia|Dec-2014	MH048671
MH048672|Malaysia|Dec-2014	MH048672
MH048673|Malaysia|Dec-2014	MH048673
MH048675|Malaysia|Dec-2014	MH048675
MH110565|China|Sep-2017	MH110565
MH110566|China|Sep-2017	MH110566
MH110567|China|Sep-2017	MH110567
MH110568|China|Sep-2017	MH110568
MH110569|China|Sep-2017	MH110569

MH110570|China|Sep-2017	MH110570
MH110571|China|Sep-2017	MH110571
MH110572|China|Sep-2017	MH110572
MH110574|China|Aug-2017	MH110574
MH110576|China|Aug-2017	MH110576
MH110577|China|Aug-2017	MH110577
MH110578|China|Aug-2017	MH110578
MH110579|China|Aug-2017	MH110579
MH110580|China|Aug-2017	MH110580
MH110581|China|Sep-2017	MH110581
MH110582|China|Sep-2017	MH110582
MH110586|China|Sep-2017	MH110586
MH110587|China|Sep-2017	MH110587
MH110588|China|Sep-2017	MH110588
MH110590|China|Sep-2017	MH110590
MH110591|China|Sep-2017	MH110591
MH110593|China|Sep-2017	MH110593
MH110594|China|Aug-2017	MH110594
MH110595|China|Sep-2017	MH110595
MH110596|China|Sep-2017	MH110596
MH110597|China|Sep-2017	MH110597
MH110598|China|Sep-2017	MH110598
MH110599|China|Sep-2017	MH110599
MH110600|China|Sep-2017	MH110600
MH110601|China|Sep-2017	MH110601
MH110602|China|Sep-2017	MH110602
MH110603|China|Aug-2017	MH110603
MH488959|Malaysia|2014	MH488959
MH823208|Indonesia|2014	MH823208
MH827526|China|2017	MH827526
MH827527|China|2017	MH827527
MH827536|China|2017	MH827536
MH827537|China|2017	MH827537
MH827539|China|2017	MH827539
MH827546|China|2017	MH827546
MH827547|China|2017	MH827547
MH827549|China|2017	MH827549
MH827550|China|2017	MH827550
MH827552|China|2017	MH827552
MH827553|China|2017	MH827553
MH827554|China|2017	MH827554
MH985858|Australia|2016	MH985858
MH985859|Australia|2017	MH985859
MK411559|Indonesia|23-Feb-2016	MK411559
MK513444|Singapore|Jan-2015	MK513444
MK543448|China|23-Sep-2018	MK543448
MK543449|China|26-Sep-2018	MK543449
MK543450|China|27-Sep-2018	MK543450
MK543471|China|23-Sep-2018	MK543471
MK543479|China|23-Sep-2018	MK543479
MK564476|China|08-Aug-2017	MK564476
MK564477|China|15-Apr-2016	MK564477
MK564478|China|22-Apr-2016	MK564478

MK564479|China|23-Aug-2016	MK564479
MK564480|China|26-Aug-2016	MK564480
MK564481|China|27-Jul-2017	MK564481
MK564482|China|07-Aug-2017	MK564482
MK564483|China|11-Aug-2017	MK564483
MK564484|China|23-Jul-2018	MK564484
MK564485|China|28-Aug-2018	MK564485
MK564486|China|28-Aug-2018	MK564486
MK564487|China|03-Sep-2018	MK564487
MK570306|DENV2|FRA-REU|2018-03	MK570306
MK570307|DENV2|FRA-REU|2018-05-15	MK570307
MK578532|China|01-Apr-2016	MK578532
MK629884|South-Korea|2015	MK629884
MK783190|China|2018	MK783190
MK783191|China|2018	MK783191
MK783192|China|2018	MK783192
MK783193|China|2018	MK783193
MK783194|China|2018	MK783194
MK783195|China|2018	MK783195
MK783196|China|2018	MK783196
MK783197|China|2018	MK783197
MK783198|China|2018	MK783198
MK783199|China|2018	MK783199
MK783200|China|2017	MK783200
MK783203|China|2018	MK783203
MK783204|China|2018	MK783204
MK783205|China|2018	MK783205
MN018337|China|20-Jun-2015	MN018337
MN018338|China|20-Aug-2015	MN018338
MN018340|China|07-Jul-2016	MN018340
MN018341|China|20-Apr-2017	MN018341
MN018342|China|23-Mar-2017	MN018342
MN018343|China|23-Mar-2017	MN018343
MN018344|China|11-Mar-2017	MN018344
MN018346|China|11-Feb-2016	MN018346
MN018347|China|10-Oct-2016	MN018347
MN018348|China|18-Jul-2016	MN018348
MN018349|China|09-May-2015	MN018349
MN018350|China|29-Jun-2016	MN018350
MN018351|China|17-May-2015	MN018351
MN018352|China|07-Sep-2015	MN018352
MN018353|China|15-Jun-2016	MN018353
MN018354|China|23-May-2017	MN018354
MN018355|China|08-May-2017	MN018355
MN018356|China|23-Sep-2014	MN018356
MN018357|China|28-May-2014	MN018357
MN018359|China|02-Jul-2017	MN018359
MN018360|China|02-Jul-2017	MN018360
MN018361|China|04-May-2017	MN018361
MN018363|China|23-Feb-2016	MN018363
MN018364|China|12-Aug-2016	MN018364
MN018365|China|30-Dec-2015	MN018365
MN272404|DENV2|FRA-REU|2018-03	MN272404

MN272405|DENV2|SYC|2016-05	MN272405
MN328061|Bangladesh|29-Jul-2019	MN328061
MN566109|New-Caledonia|01-Apr-2017	MN566109
MN566110|New-Caledonia|18-Jun-2017	MN566110
MN566111|New-Caledonia|09-Apr-2018	MN566111
MN566112|New-Caledonia|23-Apr-2018	MN566112
MN577545|Sri-Lanka|Jun-2017	MN577545
MN577547|Sri-Lanka|Jun-2017	MN577547
MN577548|Sri-Lanka|Jul-2017	MN577548
MN577549|Sri-Lanka|Aug-2017	MN577549
MN577562|Kenya|May-2013	MN577562
MN577564|Kenya|Jun-2014	MN577564
MN923107|China|15-Jun-2019	MN923107
MN923108|China|20-Jun-2019	MN923108
MN923109|China|08-Jul-2019	MN923109
MN923110|China|04-Aug-2019	MN923110
MN923111|China|24-Aug-2019	MN923111
MN923112|China|26-Aug-2019	MN923112
MN923116|China|30-Jun-2019	MN923116
MN923118|China|14-Aug-2019	MN923118
MN923119|China|21-Aug-2019	MN923119
MN923121|China|10-Oct-2019	MN923121
MN944002|China|02-Jul-2019	MN944002
MN952966|China|Nov-2015	MN952966
MN952967|China|Nov-2015	MN952967
MN982899|Australia|May-2019	MN982899
MT006136|Sri-Lanka|13-Oct-2017	MT006136
MT006137|Sri-Lanka|23-Oct-2017	MT006137
MT006138|Sri-Lanka|25-Oct-2017	MT006138
MT006139|Sri-Lanka|31-Oct-2017	MT006139
MT006140|Sri-Lanka|31-Oct-2017	MT006140
MT006141|Sri-Lanka|01-Nov-2017	MT006141
MT006142|Sri-Lanka|22-Apr-2018	MT006142
MT006143|Sri-Lanka|25-Apr-2018	MT006143
MT006144|Sri-Lanka|30-Apr-2018	MT006144
MT006145|Sri-Lanka|30-Apr-2018	MT006145
MT006146|Sri-Lanka|02-Nov-2017	MT006146
MT006148|Sri-Lanka|02-Nov-2017	MT006148
MT006149|Sri-Lanka|17-May-2018	MT006149
MT006150|Sri-Lanka|16-Nov-2017	MT006150
MT006153|Sri-Lanka|12-Nov-2017	MT006153
MT006155|Sri-Lanka|21-Nov-2017	MT006155
MT006157|Sri-Lanka|21-Nov-2017	MT006157
MT006159|Sri-Lanka|24-Nov-2017	MT006159
MT006160|Sri-Lanka|01-Dec-2017	MT006160
MT006161|Sri-Lanka|13-Dec-2017	MT006161
MT006162|Sri-Lanka|13-Dec-2017	MT006162
MT006163|Sri-Lanka|10-Jul-2018	MT006163
MT006164|Sri-Lanka|17-Jul-2018	MT006164
MT006165|Sri-Lanka|18-Jul-2018	MT006165
MT006167|Sri-Lanka|21-Dec-2017	MT006167
MT006168|Sri-Lanka|03-Jan-2018	MT006168
MT006170|Sri-Lanka|08-Jan-2018	MT006170

MT006171|Sri-Lanka|19-Jan-2018	MT006171
MT006172|Sri-Lanka|19-Jan-2018	MT006172
MT006173|Sri-Lanka|22-Jan-2018	MT006173
MT006174|Sri-Lanka|22-Jan-2018	MT006174
MT006175|Sri-Lanka|30-Jan-2018	MT006175
MT006176|Sri-Lanka|14-Feb-2018	MT006176
MT006177|Sri-Lanka|14-Mar-2018	MT006177
MT006178|Sri-Lanka|15-Mar-2018	MT006178
MT006179|Sri-Lanka|26-Mar-2018	MT006179
MT006180|Sri-Lanka|28-Mar-2018	MT006180
MT006181|Sri-Lanka|29-Mar-2018	MT006181
MT006184|Sri-Lanka|01-Apr-2018	MT006184
MT006185|Sri-Lanka|03-Apr-2018	MT006185
MT006186|Sri-Lanka|09-Apr-2018	MT006186
MT180479|Sri-Lanka|Aug-2017	MT180479
MT261956|Burkina-Faso|16-Oct-2017	MT261956
MT261957|Burkina-Faso|16-Oct-2017	MT261957
MT261958|Burkina-Faso|16-Oct-2017	MT261958
MT261959|Burkina-Faso|17-Oct-2017	MT261959
MT261960|Burkina-Faso|17-Oct-2017	MT261960
MT261961|Burkina-Faso|26-Oct-2017	MT261961
MT261962|Burkina-Faso|25-Oct-2017	MT261962
MT261963|Burkina-Faso|25-Oct-2017	MT261963
MT261964|Burkina-Faso|24-Oct-2017	MT261964
MT261965|Burkina-Faso|23-Oct-2017	MT261965
MT261966|Burkina-Faso|08-Nov-2017	MT261966
MT261967|Burkina-Faso|09-Oct-2017	MT261967
MT261968|Burkina-Faso|12-Oct-2017	MT261968
MT261969|Burkina-Faso|30-Oct-2017	MT261969
MT261970|Burkina-Faso|03-Nov-2017	MT261970
MT261971|Burkina-Faso|06-Nov-2017	MT261971
MT754367|China|24-Oct-2019	MT754367
MT754368|China|24-Oct-2019	MT754368
MT754369|China|24-Oct-2019	MT754369
MT754370|China|24-Oct-2019	MT754370
MT754371|China|24-Oct-2019	MT754371
MT832054|Philippines|2014	MT832054
MT832056|Philippines|2013	MT832056
MT832057|Philippines|2015	MT832057
MT832058|Philippines|2015	MT832058
MT832059|Philippines|2014	MT832059
MT832060|Philippines|2014	MT832060
MT832061|Philippines|2013	MT832061
MT832062|Philippines|2013	MT832062
MT832063|Philippines|2013	MT832063
MT832064|Philippines|2013	MT832064
MT832065|Philippines|2014	MT832065
MT832066|Philippines|2014	MT832066
MT832067|Philippines|2013	MT832067
MT832068|Philippines|2013	MT832068
MT832069|Philippines|2013	MT832069
MT832070|Philippines|2013	MT832070
MT832072|Philippines|2013	MT832072

MT832074|Philippines|2013	MT832074
MT832075|Philippines|2013	MT832075
MT832076|Philippines|2013	MT832076
MT832078|Philippines|2013	MT832078
MT832079|Philippines|2013	MT832079
MT832080|Philippines|2013	MT832080
MT921570|Australia|2013	MT921570
MT921571|Australia|2015	MT921571
MT921572|Australia|2000	MT921572
MT921573|Australia|2004	MT921573
MT980927|Mauritania|30-Nov-2017	MT980927
MT981011|Senegal|15-Oct-2018	MT981011
MT981085|Mauritania|13-Dec-2018	MT981085
MT981148|Senegal|04-Jan-2019	MT981148
MT982126|Burkina-Faso|10-Aug-2019	MT982126
MT982148|Burkina-Faso|08-Sep-2019	MT982148
MT982731|Burkina-Faso|02-Nov-2016	MT982731
MW186239|Singapore|Oct-2019	MW186239
MW186240|Singapore|Oct-2019	MW186240
MW288024|Senegal|Oct-2018	MW288024
MW288029|Senegal|Oct-2018	MW288029
MW288030|Senegal|Oct-2018	MW288030
MW288034|Senegal|Nov-2018	MW288034
MW295816|China|2020-08-07	MW295816
MW345921|China|2020-10-18	MW345921
MW512341|Singapore|2004	MW512341
MW512342|Singapore|2007	MW512342
MW512343|Singapore|2007	MW512343
MW512344|Singapore|2007	MW512344
MW512345|Singapore|2007	MW512345
MW512346|Singapore|2007	MW512346
MW512347|Singapore|2007	MW512347
MW512348|Singapore|2007	MW512348
MW512349|Singapore|2007	MW512349
MW512350|Singapore|2008	MW512350
MW512351|Singapore|2008	MW512351
MW512352|Singapore|2008	MW512352
MW512353|Singapore|2008	MW512353
MW512354|Singapore|2009	MW512354
MW512355|Singapore|2009	MW512355
MW512356|Singapore|2009	MW512356
MW512357|Singapore|2010	MW512357
MW512358|Singapore|2010	MW512358
MW512359|Singapore|2010	MW512359
MW512360|Singapore|2010	MW512360
MW512361|Singapore|2010	MW512361
MW512363|Singapore|2011	MW512363
MW512364|Singapore|2011	MW512364
MW512365|Singapore|2011	MW512365
MW512366|Singapore|2011	MW512366
MW512367|Singapore|2011	MW512367
MW512368|Singapore|2011	MW512368
MW512369|Singapore|2011	MW512369

MW512373|Singapore|2012	MW512373
MW512374|Singapore|2012	MW512374
MW512375|Singapore|2012	MW512375
MW512376|Singapore|2012	MW512376
MW512377|Singapore|2012	MW512377
MW512378|Singapore|2012	MW512378
MW512379|Singapore|2012	MW512379
MW512380|Singapore|2012	MW512380
MW512381|Singapore|2013	MW512381
MW512382|Singapore|2013	MW512382
MW512383|Singapore|2013	MW512383
MW512384|Singapore|2013	MW512384
MW512385|Singapore|2013	MW512385
MW512386|Singapore|2013	MW512386
MW512387|Singapore|2013	MW512387
MW512388|Singapore|2013	MW512388
MW512389|Singapore|2013	MW512389
MW512390|Singapore|2013	MW512390
MW512392|Singapore|2013	MW512392
MW512393|Singapore|2013	MW512393
MW512394|Singapore|2013	MW512394
MW512395|Singapore|2013	MW512395
MW512396|Singapore|2013	MW512396
MW512397|Singapore|2013	MW512397
MW512398|Singapore|2013	MW512398
MW512399|Singapore|2013	MW512399
MW512400|Singapore|2013	MW512400
MW512401|Singapore|2013	MW512401
MW512402|Singapore|2013	MW512402
MW512403|Singapore|2013	MW512403
MW512404|Singapore|2013	MW512404
MW512405|Singapore|2013	MW512405
MW512406|Singapore|2013	MW512406
MW512407|Singapore|2013	MW512407
MW512408|Singapore|2013	MW512408
MW512409|Singapore|2013	MW512409
MW512414|Singapore|2014	MW512414
MW512415|Singapore|2014	MW512415
MW512416|Singapore|2014	MW512416
MW512417|Singapore|2014	MW512417
MW512418|Singapore|2014	MW512418
MW512419|Singapore|2014	MW512419
MW512420|Singapore|2014	MW512420
MW512421|Singapore|2014	MW512421
MW512422|Singapore|2014	MW512422
MW512423|Singapore|2014	MW512423
MW512424|Singapore|2014	MW512424
MW512425|Singapore|2014	MW512425
MW512426|Singapore|2014	MW512426
MW512427|Singapore|2014	MW512427
MW512428|Singapore|2014	MW512428
MW512429|Singapore|2014	MW512429
MW512430|Singapore|2014	MW512430

MW512431|Singapore|2014	MW512431
MW512432|Singapore|2014	MW512432
MW512433|Singapore|2014	MW512433
MW512434|Singapore|2014	MW512434
MW512435|Singapore|2015	MW512435
MW512436|Singapore|2015	MW512436
MW512437|Singapore|2015	MW512437
MW512438|Singapore|2015	MW512438
MW512439|Singapore|2015	MW512439
MW512440|Singapore|2015	MW512440
MW512441|Singapore|2015	MW512441
MW512442|Singapore|2015	MW512442
MW512443|Singapore|2015	MW512443
MW512444|Singapore|2015	MW512444
MW512445|Singapore|2015	MW512445
MW512446|Singapore|2015	MW512446
MW512447|Singapore|2015	MW512447
MW512448|Singapore|2015	MW512448
MW512450|Singapore|2016	MW512450
MW512451|Singapore|2016	MW512451
MW512452|Singapore|2016	MW512452
MW512453|Singapore|2016	MW512453
MW512454|Singapore|2016	MW512454
MW512455|Singapore|2016	MW512455
MW512456|Singapore|2016	MW512456
MW512457|Singapore|2016	MW512457
MW512458|Singapore|2016	MW512458
MW512459|Singapore|2016	MW512459
MW512460|Singapore|2016	MW512460
MW512461|Singapore|2016	MW512461
MW512462|Singapore|2016	MW512462
MW512463|Singapore|2016	MW512463
MW512464|Singapore|2016	MW512464
MW512465|Singapore|2016	MW512465
MW512466|Singapore|2016	MW512466
MW512467|Singapore|2016	MW512467
MW512471|Singapore|2017	MW512471
MW512472|Singapore|2017	MW512472
MW512473|Singapore|2017	MW512473
MW512474|Singapore|2017	MW512474
MW512475|Singapore|2017	MW512475
MW512476|Singapore|2017	MW512476
MW512477|Singapore|2017	MW512477
MW512478|Singapore|2017	MW512478
MW512479|Singapore|2018	MW512479
MW512480|Singapore|2018	MW512480
MW512481|Singapore|2018	MW512481
MW512482|Singapore|2018	MW512482
MW512483|Singapore|2018	MW512483
MW512484|Singapore|2018	MW512484
MW512485|Singapore|2018	MW512485
MW512486|Singapore|2018	MW512486
MW512487|Singapore|2018	MW512487

MW512488|Singapore|2018	MW512488
MW512489|Singapore|2018	MW512489
MW512491|Singapore|2019	MW512491
MW512492|Singapore|2019	MW512492
MW512493|Singapore|2019	MW512493
MW512494|Singapore|2019	MW512494
MW512495|Singapore|2019	MW512495
MW512496|Singapore|2019	MW512496
MW512497|Singapore|2019	MW512497
MW512498|Singapore|2019	MW512498
MW720945|China|Sep-2019	MW720945
MW720946|China|Sep-2019	MW720946
MW720947|China|Sep-2019	MW720947
MW720950|China|Sep-2019	MW720950
MW720953|China|Sep-2019	MW720953
MW720957|China|Sep-2019	MW720957
MW721460|China|2018-10	MW721460
MW721461|China|2018-10	MW721461
MW721462|China|2014-10	MW721462
MW721463|China|2014-10	MW721463
MW721464|China|2014-10	MW721464
MW721465|China|2014-10	MW721465
MW721466|China|2014-10	MW721466
MW721467|China|2014-10	MW721467
MW721468|China|2014-10	MW721468
MW721469|China|2017-10	MW721469
MW721470|China|2017-10	MW721470
MW721471|China|2014-10	MW721471
MW721472|China|2014-10	MW721472
MW721473|China|2014-10	MW721473
MW721475|China|2010-10	MW721475
MW730814|Nepal|Jul-2017	MW730814
MW730815|Nepal|Jul-2017	MW730815
MW730816|Nepal|Jul-2017	MW730816
MW730817|Nepal|Jul-2017	MW730817
MW730818|Nepal|Jul-2017	MW730818
MW730819|Nepal|Jul-2017	MW730819
MW730820|Nepal|Jul-2017	MW730820
MW730821|Nepal|Jul-2017	MW730821
MW730822|Nepal|Jul-2017	MW730822
MW730823|Nepal|Jul-2017	MW730823
MW730824|Nepal|Jul-2017	MW730824
MW730825|Nepal|Jul-2017	MW730825
MW730826|Nepal|Jul-2017	MW730826
MW730827|Nepal|Jul-2017	MW730827
MW730828|Nepal|Jul-2017	MW730828
MW730829|Nepal|Jul-2017	MW730829
MW730830|Nepal|Jul-2017	MW730830
MW730831|Nepal|Jul-2017	MW730831
MW730832|Nepal|Jul-2017	MW730832
MW730833|Nepal|Jul-2017	MW730833
MW730839|Nepal|Jul-2017	MW730839
MW881533|China|2021-02-02	MW881533

MW945435|Viet-Nam|2006	MW945435
MW946584|Thailand|2007	MW946584
MZ130519|Mozambique|2019-03-05	MZ130519
MZ130522|Egypt|2017-11-07	MZ130522
MZ130524|Benin|2019-07-09	MZ130524
MZ130525|Cameroon|2019-12-29	MZ130525
MZ130526|Tanzania|2014-05-21	MZ130526
MZ453006|China|2019	MZ453006
MZ453008|China|2019	MZ453008
MZ453009|China|2019	MZ453009
MZ453010|China|2019	MZ453010
MZ453011|China|2019	MZ453011
MZ636802|Thailand|24-Nov-2019	MZ636802
MZ636803|Thailand|28-Nov-2019	MZ636803
MZ636804|Thailand|30-Nov-2019	MZ636804
MZ636805|Thailand|30-Dec-2019	MZ636805
MZ857212|Tanzania|2014	MZ857212
MZ857213|Tanzania|2014-06	MZ857213
MZ857214|Tanzania|2014	MZ857214
MZ857215|Somalia|2015	MZ857215
MZ857220|Burkina-Faso|2017	MZ857220
OK469352|Singapore|1993	OK469352
OK559627|China|26-Sep-2019	OK559627
OL321176|Papua-New-Guinea|2016	OL321176
OL321177|Papua-New-Guinea|2016	OL321177
OL321178|Papua-New-Guinea|07-Mar-2016	OL321178
OL321182|Papua-New-Guinea|05-Mar-2016	OL321182
OL321183|Papua-New-Guinea|12-Mar-2016	OL321183
OL321184|Papua-New-Guinea|10-May-2016	OL321184
OL321185|Papua-New-Guinea|2016	OL321185
OL321186|Papua-New-Guinea|12-Apr-2016	OL321186
OL412740|Cambodia|Jul-2019	OL412740
OL414717|Cambodia|Jul-2019	OL414717
OL414722|Cambodia|Aug-2019	OL414722
OL414723|Cambodia|Aug-2019	OL414723
OL414724|Cambodia|Aug-2019	OL414724
OL414725|Cambodia|Aug-2019	OL414725
OL414726|Cambodia|Aug-2019	OL414726
OL414727|Cambodia|Aug-2019	OL414727
OL414730|Cambodia|Jul-2019	OL414730
OL414732|Cambodia|Aug-2020	OL414732
OL414733|Cambodia|Aug-2020	OL414733
OL414737|Cambodia|Jun-2019	OL414737
OL414738|Cambodia|Jun-2019	OL414738
OL414740|Cambodia|Jun-2019	OL414740
OL414741|Cambodia|Jul-2019	OL414741
OL414742|Cambodia|Jun-2019	OL414742
OL414745|Cambodia|Aug-2019	OL414745
OL414746|Cambodia|Jun-2020	OL414746
OL414747|Cambodia|Aug-2019	OL414747
OL414749|Cambodia|Jun-2020	OL414749
OL414751|Cambodia|Jul-2020	OL414751
OL414752|Cambodia|Jul-2020	OL414752

OL414753|Cambodia|Aug-2020	OL414753
OL414754|Cambodia|Aug-2020	OL414754
OL414755|Cambodia|Aug-2020	OL414755
OL414756|Cambodia|Aug-2020	OL414756
OL414758|Cambodia|Sep-2020	OL414758
OL414760|Cambodia|Aug-2019	OL414760
OL414762|Cambodia|Jul-2019	OL414762
OL414763|Cambodia|Aug-2019	OL414763
OL414764|Cambodia|Aug-2020	OL414764
OL414765|Cambodia|Aug-2020	OL414765
OL420733|Cambodia|Jul-2019	OL420733
OL435143|Cambodia|Aug-2020	OL435143
OM317565|Cameroon|24-Jun-2020	OM317565
OM317566|Cameroon|02-Aug-2020	OM317566
OM349569|China|25-Sep-2019	OM349569
OM368351|China|16-Sep-2019	OM368351
OM368352|China|17-May-2018	OM368352
OM639979|India|19-Oct-2021	OM639979
OM639980|India|16-Nov-2021	OM639980
OM639982|India|14-Sep-2021	OM639982
OM639984|India|16-Nov-2021	OM639984
OM639992|India|16-Nov-2021	OM639992
OM639993|India|16-Nov-2021	OM639993
OM680963|India|16-Nov-2021	OM680963
OM700180|India|16-Nov-2021	OM700180
OM730078|India|16-Nov-2021	OM730078
OM744143|Brazil|2021-11-29	OM744143
OM791800|Peru|26-Sep-2019	OM791800
OM791801|Peru|28-Sep-2019	OM791801
ON123638|Peru|01-Mar-2021	ON123638
ON123639|Peru|04-Mar-2021	ON123639
ON123640|Peru|08-Mar-2021	ON123640
ON123641|Peru|24-Mar-2021	ON123641
ON123642|Peru|08-Apr-2021	ON123642
ON123643|Peru|12-Apr-2021	ON123643
ON123644|Peru|21-Jul-2021	ON123644
ON231304|Senegal|26-Oct-2018	ON231304
ON231305|Senegal|29-Oct-2018	ON231305
ON231306|Senegal|28-Oct-2018	ON231306
ON231307|Senegal|28-Oct-2018	ON231307
ON231308|Senegal|28-Oct-2018	ON231308
ON231309|Senegal|28-Oct-2018	ON231309
ON231310|Senegal|31-Oct-2018	ON231310
ON231311|Senegal|09-Nov-2018	ON231311
ON231312|Senegal|08-Nov-2018	ON231312
ON231313|Senegal|09-Nov-2018	ON231313
ON634742|Brazil|05-Feb-2022	ON634742
ON634743|Brazil|15-Mar-2022	ON634743
ON634745|Brazil|14-Mar-2022	ON634745
ON634750|Brazil|24-Mar-2022	ON634750
ON634755|Brazil|06-Mar-2022	ON634755
ON634756|Brazil|30-Jan-2022	ON634756
ON875316|China|17-May-2018	ON875316

ON885253|Philippines|02-Jul-2019	ON885253
ON885286|Niger|15-Jun-2019	ON885286
ON890351|China|07-Aug-2017	ON890351
ON908222|China|17-May-2018	ON908222
ON908223|Niger|15-Jun-2019	ON908223
ON908224|Cambodia|08-Jul-2019	ON908224
ON908226|China|28-Aug-2018	ON908226
ON908228|China|07-Aug-2017	ON908228
ON908229|China|16-Sep-2019	ON908229
OP389112|China|25-Jul-2022	OP389112
OP410989|Singapore|Jul-2007	OP410989
OP410990|Singapore|Oct-2019	OP410990
OP410992|Singapore|Oct-2016	OP410992
OP684143|China|12-Sep-2017	OP684143
OP684144|China|18-Sep-2017	OP684144
OP684145|China|20-Sep-2017	OP684145
OP684147|China|19-Sep-2017	OP684147
OP684148|China|21-Sep-2017	OP684148
OP684150|China|27-Sep-2017	OP684150
OP684151|China|26-Sep-2017	OP684151
OP684152|China|02-Jul-2017	OP684152
OP684155|China|28-Aug-2017	OP684155
OP684156|China|22-Aug-2017	OP684156
OP684157|China|30-Aug-2017	OP684157
OP684158|China|29-Aug-2017	OP684158
OP684159|China|31-Aug-2017	OP684159
OP684163|China|29-Aug-2017	OP684163
OP684164|China|29-Aug-2017	OP684164
OP684165|China|01-Sep-2017	OP684165
OP684167|China|02-Sep-2017	OP684167
OP684168|China|03-Sep-2017	OP684168
OP684170|China|05-Sep-2017	OP684170
OP684172|China|06-Sep-2017	OP684172
OP684173|China|05-Sep-2017	OP684173
OP684174|China|05-Sep-2017	OP684174
OP684175|China|05-Sep-2017	OP684175
OP684176|China|05-Sep-2017	OP684176
OP684177|China|07-Sep-2017	OP684177
OP684178|China|11-Sep-2017	OP684178
OP684179|China|12-Sep-2017	OP684179
OP684180|China|02-Jul-2018	OP684180
OP684181|China|03-Jul-2018	OP684181
OP684182|China|01-Aug-2018	OP684182
OP684183|China|27-May-2018	OP684183
OP684186|China|28-May-2018	OP684186
OP684187|China|07-Sep-2018	OP684187
OP684194|China|16-Jun-2018	OP684194
OP684195|China|20-Jun-2018	OP684195
OP684196|China|05-Jan-2019	OP684196
OP684198|China|20-Jan-2019	OP684198
OP684199|China|31-Jul-2019	OP684199
OP684200|China|08-Aug-2019	OP684200
OP684201|China|16-Aug-2019	OP684201

OP684204|China|17-Sep-2019	OP684204
OP684205|China|28-Sep-2019	OP684205
OP684206|China|03-Oct-2019	OP684206
OP684208|China|06-Oct-2019	OP684208
OP684209|China|12-Oct-2019	OP684209
OP684210|China|15-Oct-2019	OP684210
OP684213|China|14-Nov-2019	OP684213
OP684214|China|30-Nov-2019	OP684214
OP684216|China|11-Mar-2019	OP684216
OP809582|India|2021	OP809582
OP811977|Pakistan|27-Apr-2022	OP811977
OP811978|Pakistan|27-Apr-2022	OP811978
OP811979|Pakistan|28-Apr-2022	OP811979
OP811980|Pakistan|28-Apr-2022	OP811980
OP811981|Pakistan|18-Oct-2022	OP811981
OP811982|Pakistan|13-Sep-2022	OP811982
OP811983|Pakistan|28-Sep-2022	OP811983
OP811984|Pakistan|30-Sep-2022	OP811984
OP895917|Maldives|Dec-2021	OP895917
OP895918|Cambodia|Apr-2019	OP895918
OP895919|Malaysia|Nov-2018	OP895919
OP898559|Pakistan|Oct-2022	OP898559
OP921000|India|29-Jul-2022	OP921000
OP921001|India|27-Jul-2022	OP921001
OP941832|Brazil|2022-10-11	OP941832
OP941834|Brazil|2022-11-02	OP941834
OP941835|Brazil|2022-11-04	OP941835
OP941836|Brazil|2022-11-01	OP941836
OP941838|Brazil|2022-10-19	OP941838
OP941840|Brazil|2022-10-24	OP941840
OP941841|Brazil|2022-11-03	OP941841
OP941842|Brazil|2022-10-18	OP941842
OP941843|Brazil|2022-10-18	OP941843
OP941844|Brazil|2022-11-06	OP941844
OP984834|Viet-Nam|20-Nov-2022	OP984834
OP999336|Cambodia|Sep-2020	OP999336
OP999339|Cambodia|Sep-2020	OP999339
OQ000263|Cambodia|Sep-2020	OQ000263
OQ028216|Viet-Nam|25-May-2022	OQ028216
OQ028217|Viet-Nam|07-Jun-2019	OQ028217
OQ028220|Viet-Nam|14-Aug-2019	OQ028220
OQ028225|Viet-Nam|23-Sep-2020	OQ028225
OQ028227|Viet-Nam|31-Jul-2019	OQ028227
OQ028230|Viet-Nam|07-Sep-2020	OQ028230
OQ028232|Viet-Nam|02-Mar-2020	OQ028232
OQ426766|Viet-Nam|09-Jan-2019	OQ426766
OQ426773|Viet-Nam|22-Oct-2018	OQ426773
OQ426782|Viet-Nam|20-Dec-2018	OQ426782
OQ426847|Viet-Nam|18-Dec-2018	OQ426847
OQ426916|Viet-Nam|10-Sep-2018	OQ426916
OQ426918|Viet-Nam|19-Sep-2018	OQ426918
OQ622206|Brazil|02-Apr-2022	OQ622206
OQ622207|Brazil|18-Mar-2022	OQ622207

OQ653841|China|22-Jul-2022	OQ653841
OQ674509|Cambodia|08-Sep-2019	OQ674509
OQ678017|Cambodia|30-Oct-2019	OQ678017
OQ678059|Cambodia|31-Oct-2019	OQ678059
OQ678101|Cambodia|03-Jun-2020	OQ678101
OQ678102|Cambodia|30-Jul-2020	OQ678102
OQ683881|Cambodia|02-Jun-2020	OQ683881
OQ786047|Brazil|18-Jan-2023	OQ786047
OQ786048|Brazil|22-Jan-2023	OQ786048
OQ821481|India|2022-07-01	OQ821481
OQ821482|India|2022-08-19	OQ821482
OQ821493|Philippines|2019-07-31	OQ821493
OR025600|Brazil|23-Mar-2023	OR025600
OR025669|Brazil|27-Mar-2023	OR025669
OR025670|Brazil|27-Mar-2023	OR025670
OR025672|Brazil|05-Mar-2023	OR025672
OR025674|Brazil|14-Mar-2023	OR025674
OR025675|Brazil|16-Mar-2023	OR025675
OR025676|Brazil|19-Apr-2023	OR025676
OR029721|China|16-Aug-2019	OR029721
OR029722|China|17-Aug-2019	OR029722
OR029723|China|17-Aug-2019	OR029723
OR029728|China|20-Aug-2019	OR029728
OR029729|China|20-Aug-2019	OR029729
OR029730|China|20-Aug-2019	OR029730
OR029732|China|21-Aug-2019	OR029732
OR029734|China|22-Aug-2019	OR029734
OR029735|China|22-Aug-2019	OR029735
OR029737|China|06-Aug-2019	OR029737
OR029738|China|12-Aug-2019	OR029738
OR029740|China|15-Aug-2019	OR029740
OR029741|China|17-Aug-2019	OR029741
OR039496|Brazil|2022-03-24	OR039496
OR039497|Brazil|2022-03-28	OR039497
OR039498|Brazil|2022-03-30	OR039498
OR039499|Brazil|2022-03-30	OR039499
OR039500|Brazil|2022-03-28	OR039500
OR039501|Brazil|2022-04-01	OR039501
OR039502|Brazil|2022-04-01	OR039502
OR039503|Brazil|2022-04-02	OR039503
OR039504|Brazil|2022-04-02	OR039504
OR039505|Brazil|2022-04-01	OR039505
OR039506|Brazil|2022-04-03	OR039506
OR039507|Brazil|2022-04-13	OR039507
OR039508|Brazil|2022-04-22	OR039508
OR039509|Brazil|2022-03-08	OR039509
OR039510|Brazil|2022-03-08	OR039510
OR039511|Brazil|2022-04-18	OR039511
OR039513|Brazil|2022-04-01	OR039513
OR039514|Brazil|2022-04-05	OR039514
OR039515|Brazil|2022-04-11	OR039515
OR039516|Brazil|2022-04-18	OR039516
OR039517|Brazil|2022-05-08	OR039517

OR039518|Brazil|2023-03-12	OR039518
OR125606|China|06-May-2023	OR125606
OR771147|USA|2023-7-20	OR771147
OR771188|USA|2023-9-26	OR771188
OR804009|Guadeloupe|2023-04	OR804009
OR821962|USA|2023-10-11	OR821962
